# Development of a reliable empirical correlation to calculate hydrogen solubility in seventeen alcoholic media

**DOI:** 10.1038/s41598-022-13720-1

**Published:** 2022-06-10

**Authors:** Saleh Hosseini

**Affiliations:** Department of Chemical Engineering, University of Larestan, Larestan, Iran

**Keywords:** Chemical engineering, Chemistry

## Abstract

This study uses the differential evolution optimization algorithm to adjust the coefficient of Arrhenius-shape correlation for calculating hydrogen (H_2_) solubility in alcohol-based media. The pre-exponential and exponential parts of this correlation are the functions of pressure and absolute temperature, respectively. Since this model has been validated using seventeen alcohol/hydrogen binary mixtures, it is the most generalized correlation in this regard. The proposed Arrhenius-shape correlation predicts 285 laboratory solubility measurements with the absolute average relative deviation (AARD%) of 3.28% and regression coefficient (R^2^) of 0.99589. The accuracy of the developed model has also been compared with two empirical correlations and three equations of state suggested in the literature. The Arrhenius-shape model has 15% and 50% smaller AARD than the most accurate empirical correlation and equation of state, respectively. Simulation findings demonstrate that all alcohol/hydrogen mixtures thermodynamically behave based on Henry’s law. Hydrogen solubility in alcohols increases by increasing either pressure or temperature. 1-octanol has the maximum ability to absorb the H_2_ molecules.

## Introduction

In recent decades, concerns about energy security have been raised due to the depletion of hydrocarbon reserves^1^ and the dramatically increasing demand for energy^2,3^ and fossil-based fuels^4–6^. Furthermore, the environmental problems associated with fossil fuel consumption^7^, such as gas emissions^8^, climate change^9^, and global warming^10^ have highlighted this concern further. In this regard, renewable and clean energy like hydrogen^11^ and ethanol^12^ fuels, solar irradiation^13,14^, and biomass^15,16^ have gained significant interest in reducing the dependency on fossil-derived fuels and their related environmental problems^17,18^.

Meanwhile, the construction of large-scale biorefineries still encounters enormous challenges, such as cellulose conversion efficiency and economic justification^17^. Furthermore, the bio-based feedstocks’ low heating value, high viscosity, and chemical instability should be resolved^19^. The presence of molecules with oxygen-containing groups such as alcohols, carboxylic acid, and aldehydes is responsible for these limitations^20^. Therefore, the hydrotreating/hydrodeoxygenation unit is included to remove oxygen from these oxygenated compounds^21^. In this catalytic unit, the bio-based feedstocks are processed under hydrogen pressure (7–20 MPa) at a high temperature (573.15–723.15 K)^20,22^.

Estimating the hydrogen solubility in oxygenated materials using a valid thermodynamic model is a prerequisite for feasible study, designing, optimizing, and controlling the hydrotreating/hydrodeoxygenation process^20^. Accurate measuring or reliable estimating of the hydrogen solubility in the considered liquid media has a central role in the appropriate design of hydro-processing units^22,23^.

Furthermore, hydrogen solubility in liquid media is a fundamental thermodynamic characteristic in investigating separation^24^ and conversion^25^ efficiency. For instance, methanol/hydrogen phase equilibria are required to calculate H_2_ losses by co absorption from syngas^24^. Alcohol/hydrogen and hydrogen-enriched blends may be a replacement for fossil fuel in spark-ignition engines^26^. Hydrogen production from the alcohol-based reforming^27^, including methanol^28^ and ethyl alcohol^29^, is also affected by the hydrogen solubility behavior. Moreover, the hydrogen production using the photocatalytic^30^, hydrogen-liquid-hydride slurry^31^, and aqueous and anhydrous methanol^32^ reactors is also controlled by H_2_/liquid phase behavior. Reliable knowledge about hydrogen solubility in bio-based alcohols (ethanol and glycerol) is necessary for hydrogen production using dehydrogenation reactions^33,34^. Aldehydes conversion into alcohols also occurs under the hydrogen pressure as a gaseous stream^35^.

Some researchers have experimentally measured H_2_ solubility in different alcohols, including methanol^24,36,37^, ethanol^36^, 1,2-ethanediol^38^, 1-propanol^36^, 2-propanol^39^, 2-methoxy ethanol^38^, allyl alcohol^40^, 1-butanol^36^, 2-butanol^38^, isobutanol^38^, 2-ethoxyethanol^38^, furfuryl alcohol^22^, 1-pentanol^38^, 1-hexanol^38^, 2-butoxyethanol^38^, 1-octanol^31^, and 2-ethyl hexanol-l^38^. These experimental researches have been comprehensively analyzed in the succeeding parts of the article (see “Experimental measurement of alcohol/hydrogen binary mixtures” section). Moreover, several thermodynamic^22,39,40^, empirical^41^, and intelligent^42^ paradigms have been developed for estimating the hydrogen solubility/storage in different liquid or solid media. The literature only employed some well-known equations of states, i.e., the PC-SAFT (perturbed-chain statistically associating fluid theory), PR (Peng-Robinson), and SRK (Soave–Redlich–Kwong) to estimate hydrogen dissolution in some limited alcoholic media, i.e., 2-propanol/hydrogen^39^, furfuryl alcohol/hydrogen^22^, and allyl alcohol/hydrogen^40^. The thermodynamic-based scenarios often need high computational efforts, are valid for specific alcohol/hydrogen media, and sometimes provide a relatively high uncertainty level^20^. On the other hand, the available empirical correlations in the literature are only valid for calculating hydrogen solubility in primary alcohols with up to four carbon atoms^41^. Although the empirical correlations are fast enough, they suffer from the generalization ability and provide relatively high errors^41^. The available intelligent approaches are specifically usable for estimating hydrogen solubility in furfural alcohol^23^ and primary alcohols with a maximum of four carbon atoms^41^. Therefore, developing a straightforward, simple, and computationally efficient model for estimating hydrogen solubility in a wide range of alcoholics seems inevitable.

The literature provides no general approach for accurately predicting hydrogen solubility in alcoholic compounds. Therefore, the current study proposes an Arrhenius-shape correlation to calculate H_2_ solubility in seventeen alcoholic solvents. This correlation is assembled in the simplest form, only has three parameters, and solely needs pressure and temperature to provide the hydrogen dissolution value in alcohols. Furthermore, this Arrhenius-shape model is more generalized than the empirical correlations and equations of state suggested in the literature, and it also outperforms them by more than 50% and 15%, respectively.

## Methods

Different types of mathematical scenarios^43,44^, such as predictive approach^45–48^, empirical/semi-empirical^49,50^, wavelet transform^3,51^, fuzzy model^52^, support vector machines^53^, artificial neural network^54–57^, dynamic modeling^58^, and numerical simulation^59–62^ have been extracted from historical data of considered problems. This study develops a three-parameter Arrhenius-shape correlation to estimate the hydrogen dissolution in various alcohols. The unknown coefficient of this correlation adjusts using the differential evolution (DE) optimization algorithm. Therefore, this section concisely reviews the mathematical formulations of the Arrhenius correlation and DE optimization algorithm. After that, the gathered experimental data for hydrogen dissolution in alcohols are presented and analyzed. Finally, some statistical indices for monitoring the prediction uncertainty of the Arrhenius-shape correlation have been introduced.

### Arrhenius correlation

It is obvious that a pure gas solubility in a pure liquid (like hydrogen solubility in pure alcohol) is only a function of pressure and temperature^39^. This study employs the Arrhenius-shape correlation to relate the hydrogen solubility in alcohols to the pressure and temperature^63^. Equation (1) presents a general form of the Arrhenius correlation^63^.

1 $$\gamma \, = \,\gamma_{0} \,\exp \,\left( { - \,\frac{{E_{a} /R}}{\,T}} \right)$$

In this equation, $\gamma$ shows the dependent variable, $\gamma_{0}$ is the pre-exponential term, $E_{a}$ represents activation energy, T designates the absolute temperature, and R stands for the gas constant.

The trial-and-error process confirms that the most accurate predictions for hydrogen solubility in alcoholic media are obtained when the pre-exponential and exponential parts are functions of pressure and temperature, respectively. Indeed, the pre-exponential term linearly relates to the pressure, and the exponential part is solely temperature-dependent.

### Differential evolution algorithm

The considered Arrhenius-shape correlation has three coefficients that are needed to be adjusted using an efficient optimization scenario (see “Development phase” section). The DE categorizes as a population-based algorithm for locating the global optimization condition^64^. Four central stages are followed in the DE algorithm for finding the global optima, i.e., generating initial population, mutation, crossover, and selection^64^. Equation (2) defines as a general form of an optimization problem^64^.

2 $$\begin{aligned} & minimize\,\,\,\,\,\,\,\,\,\,\,\,\,\,\,\,\,\,\,\,OF\left( X \right) \\ & with\,respect\,to:\quad \left\{ {\begin{array}{*{20}l} {X_{LB} \le X \le X_{UB} } \hfill & {} \hfill \\ {g_{i} \left( X \right) \le 0} \hfill & {i = 1,2, \ldots ,m} \hfill \\ \end{array} } \right. \\ \end{aligned}$$

where $OF\left( X \right)$ is an objective function, $X = \left( {x_{1} ,x_{2} ,......x_{n} } \right)^{T}$ indicates the decision vector with n design variables, and $g_{i} \left( X \right)$ shows equality and inequality constraints. The LB and UB subscripts are lower-bound and upper-bound of the feasible domain.

The optimization process by the DE algorithm begins with the generation of an initial population with specific numbers of random vectors (i.e., NP) in the feasible domain. Then, mutant vectors ($\mu_{i}$) are generated for all randomly generated vectors in the initial population (or previous generation) using Eq. (3).

3 $$\mu_{i}^{\,G + 1} = X_{{R_{1} }}^{G} + \varphi \left( {X_{{R_{2} }}^{G} - X_{{R_{3} }}^{G} } \right)\,\,\left\{ {\begin{array}{*{20}l} {i = 1,2,\,...\,\,,\,NP} \hfill \\ {R_{1} \, \ne \,R_{2} \, \ne \,R_{3} \, \ne i} \hfill \\ {G = 0,1,2,\,...\,,\,G^{\max } } \hfill \\ \end{array} } \right.$$

here, G shows the number of generations or optimization iterations (G = 0 indicates the initial population). Furthermore, R_1_, R_2_, and R_3_ are randomly selected integers ranging from one to NP. Finally, $\varphi$ is a positive value between zero and two that controls the effect of $X_{{R_{2} }}^{G}$-$X_{{R_{3} }}^{G}$ on the mutant vector.

The crossover scenario is then employed to enhance the diversity of the trial vector ($\psi$) by mixing the original ($X_{ji}^{G}$) and mutant ($\mu_{ji}^{G + 1}$) vectors based on Eq. (4).

4 $$\psi_{ji}^{G + 1} \, = \,\left\{ {\begin{array}{*{20}l} {\mu_{ji}^{G + 1} } \hfill & {if\,(rand(j)\, \le \,\kappa )\,or\,j = rand\,(i)} \hfill \\ {X_{ji}^{G} } \hfill & {otherwise} \hfill \\ \end{array} } \right.\quad j\, = \,1,\,2,\,...,\,n$$

here, the crossover (i.e., the value between zero and one) shows by $\kappa$. $rand(j)$ is the jth member of a randomly produced vector with values in the range of [0 1]. $rand\,(i)$ is a random integer value between one and dimension of the decision vector.

Finally, the fourth stage compares the objective function values obtained by the original ($X_{ji}^{G}$) and trial vector ($\psi$) vectors and decides which one should attend at the next generation (Eq. 5).

5 $$X_{i}^{G + 1} \, = \,\left\{ {\begin{array}{*{20}l} {X_{i}^{G} } \hfill & {if{:}\,\,\,\,OF\left( {X_{i}^{G} } \right) \le OF\left( {\psi_{i}^{G + 1} } \right)} \hfill \\ {\psi_{i}^{G + 1} } \hfill & {if{:}\,\,\,\,OF\left( {X_{i}^{G} } \right)\, > \,OF\left( {\psi_{i}^{G + 1} } \right)} \hfill \\ \end{array} } \right.$$

This process is performed on all decision vectors in all generations. The optimization continues until the maximum number of iterations is reached.

In summary, the differential evolution algorithm needs OF (objective function), g (constraints), feasible region, NP (numbers of initial population), $G^{\max }$ (numbers of iteration), $\varphi$ (mutation constant), and $\kappa$ (crossover factor) to do its duty.

The objective function of the current study is the deviation between experimental hydrogen solubility data and their associated predictions by the Arrhenius-shape correlation. The DE algorithm adjusts the unknown coefficients of the proposed model by minimizing this objective function.

### Experimental measurement of alcohol/hydrogen binary mixtures

This study checks a broad range of alcohol/hydrogen binary mixtures to approve the generalization feature of the developed Arrhenius-shape correlation. To do so, a databank of laboratory measurements for hydrogen solubility in seventeen alcohols (i.e., methanol, ethanol, 1,2-ethanediol, 1-propanol, 2-propanol, 2-methoxy ethanol, allyl alcohol, 1-butanol, 2-butanol, isobutanol, 2-ethoxyethanol, furfuryl alcohol, 1-pentanol, 1-hexanol, 2-butoxyethanol, 1-octanol, and 2-ethyl hexanol-l) has been collected from eight different references^22,24,31,36–40^.

Table 1 reports the ranges of equilibrium conditions (pressure and temperature) and hydrogen solubility data reported in the literature^22,24,31,36–40^. This table also presents the sources of experimental data and the number of measurements for each alcohol/hydrogen binary mixture. Cumulatively, 285 laboratory measurements for hydrogen solubility in seventeen alcoholic solvents have been gathered. This experimental databank is used to adjust the unknown coefficient of the Arrhenius-shape correlation and compare its performance with the available equations of state and empirical correlations.

**Table 1:** Summary of laboratory-measured phase equilibria of alcohol/hydrogen mixtures.

H_2_ (2) +	Pressure range (MPa)	Temperature range (K)	Solubility range (mole fraction)	Number of data
Methanol^24,36,37^	0.0404–43.150	248.41–476.60	0.00004–0.06130	57
Ethanol^36^	3.7000–9.0300	299.90–476.50	0.01320–0.07040	18
1,2-Ethanediol^38^	2.5300–8.9700	298.15–373.15	0.00100–0.00539	12
1-Propanol^36^	3.6400–10.190	299.90–513.60	0.01110–0.10520	25
2-Propanol^39^	5.3000–12.470	323.40–476.10	0.01390–0.06370	11
2-Methoxy ethanol^38^	2.7250–10.100	298.15–373.15	0.00490–0.02117	10
Allyl alcohol^40^	4.4000–15.250	341.50–473.00	0.01430–0.06160	21
1-Butanol^36^	3.9100–8.8200	295.30–524.90	0.01370–0.10640	11
2-Butanol^38^	1.9450–9.9650	298.15–373.15	0.00750–0.04190	10
Isobutanol^38^	2.6150–9.7200	298.15–373.15	0.00740–0.03540	7
2-Ethoxyethanol^38^	2.3900–10.820	298.15–373.15	0.00524–0.03260	12
Furfuryl alcohol^22^	5.1970–26.348	323.15–423.15	0.00690–0.06170	39
1-Pentanol^38^	2.5400–9.8000	298.15–373.15	0.00800–0.03330	10
1-Hexanol^38^	3.0100–10.340	298.15–373.15	0.00995–0.04782	14
2-Butoxyethanol^38^	3.4400–10.270	298.15–373.15	0.01207–0.03815	12
1-Octanol^31^	0.6895–1.3790	295.15–295.15	0.00246–0.00541	5
2-Ethyl hexanol-l^38^	1.9720–9.7800	298.15–393.15	0.00758–0.05240	11

### Model evaluation

Several visual and numerical analyses have been done to investigate the capability of the proposed Arrhenius-shape correlation for simulating the phase equilibrium of different alcohol/hydrogen binary systems (see “Assessment phase” section). The analyses are performed using six well-known statistical matrices, i.e., AARD%, average absolute errors (AAE), average square errors (ASE), relative absolute deviation (RAD%), relative deviation (RD%), and R^2^ (Eqs. 6–11).

6 $$AARD\% = \frac{100}{N}\sum\limits_{k = 1}^{N} {\left( {\left| {x_{2}^{act} - x_{2}^{pred} } \right|/x_{2}^{act} } \right)}_{k}$$

7 $$AAE = \,\frac{1}{N}\,\sum\limits_{k = 1}^{N} {\left| {x_{2}^{act} - x_{2}^{pred} } \right|}_{k}$$

8 $$RAD\% = \,\,100\, \times \sum\limits_{k = 1}^{N} {\left| {x_{2}^{act} - x_{2}^{pred} } \right|_{k} /\sum\limits_{k = 1}^{N} {\left| {x_{2}^{act} - \overline{{x_{2}^{act} }} } \right|_{k} } }$$

9 $$ASE = \frac{1}{N}\sum\limits_{k = 1}^{N} {\left( {x_{2}^{act} - x_{2}^{pred} } \right)_{k}^{2} }$$

10 $$RD = \left( {x_{2}^{act} - x_{2}^{pred} } \right)_{k}$$

11 $$R^{2} = 1 - \,\left\{ {\sum\limits_{k = 1}^{N} {\left( {x_{2}^{act} - x_{2}^{pred} } \right)_{k}^{2} /\sum\limits_{k = 1}^{N} {\left( {x_{2}^{act} - \overline{{x_{2}^{act} }} } \right)_{k}^{2} } } } \right\}$$

The number of data (N), the actual value of hydrogen solubility ($x_{2}^{act}$), its average value ($\overline{{x_{2}^{act} }}$), and predicted data ($x_{2}^{pred}$) are required to quantize the model’s uncertainty. These statistical indices are applied over the predictions of the equations of state, the Arrhenius-shape model, and other empirical correlations suggested in the literature.

## Results and discussion

This section introduces the final form of the Arrhenius-shape correlation and compares its prediction accuracy with the empirical correlations and equations of state available in the literature. Finally, the effect of equilibrium pressure, temperature, and alcohol type on hydrogen dissolution is investigated using the proposed correlation and from the experimental perspective.

### Development phase

It is noted that this study aims to develop an Arrhenius-shape correlation for calculating the hydrogen solubility in a wide range of alcoholic solvents. The trial-and-error analysis approved that the pre-exponential term of the Arrhenius correlation is only a function of pressure (P), while its exponential part is temperature-dependent (T). The mathematical formulation of this statement is expressed by Eq. (12).

12 $$x_{2}^{perd.} \, = \,\left( {\alpha_{z} \,P\, + \,\beta_{z} \,} \right)\, \times \,\exp \,\left( { - \frac{{\lambda_{z} }}{T}} \right)\quad z = 1,\,2,\,...,\,17$$

In the above equation, $\alpha$, $\beta$, and $\lambda$ are coefficients of the Arrhenius-shape correlation that adjust by the differential evolution optimization algorithm. The objective function is the absolute average relative deviation between experimental hydrogen solubility data and their prediction values by the Arrhenius-shape correlation (Eq. 6). The numerical values of user-entry parameters for the DE optimization algorithm have been reported in Table 2.

**Table 2:** Entry parameters for the DE optimization algorithm.

DE parameter	$G^{\max }$	n	NP	$\kappa$	$\varphi$
Entry value	1000	3	200	1	0.8

The adjusted coefficients of the Arrhenius-shape correlation for different alcohol/hydrogen mixtures have been reported in Table 3. The positive values of $\alpha$ and $\lambda$ for all binary mixtures approve that hydrogen solubility increases by increasing either pressure or temperature (or both).

**Table 3:** Adjusted coefficients of Eq. (12) for different H_2_/alcohol binary mixtures.

H_2_ (2) +	$\alpha$ (1/MPa)	$\beta$ (–)	$\lambda$ (K)
Methanol	0.029876	− 0.000180	856.1840
Ethanol	0.023976	0.256236	941.6110
1,2-Ethanediol	0.004193	− 0.000220	707.4406
1-Propanol	0.047631	0.066003	917.3786
2-Propanol	0.019380	− 0.011430	608.6667
2-Methoxy ethanol	0.020878	− 0.000270	800.5288
Allyl alcohol	0.034288	− 0.036260	917.2720
1-Butanol	0.025247	0.409363	992.5810
2-Butanol	0.026317	0.002606	688.0561
Isobutanol	0.018566	0.004370	582.8059
2-Ethoxyethanol	0.020440	0.006338	701.9819
Furfuryl alcohol	0.016136	0.005666	823.8483
1-Pentanol	0.019202	0.002633	574.0499
1-Hexanol	0.020440	0.003970	564.4754
2-Butoxyethanol	0.025400	0.001278	644.3644
1-Octanol	0.080728	− 0.009250	867.0004
2-Ethyl hexanol-l	0.026627	0.007362	599.5861

Table 3 also states that the Arrhenius-shape correlation is applicable for modeling the phase equilibria of seventeen alcohol/hydrogen solutions. This is the most generalized empirical correlation developed for estimating phase equilibria of alcohol/hydrogen solutions up to now.

### Assessment phase

This section focuses on validating the Arrhenius-shape model by laboratory-measured hydrogen solubility data, empirical correlations, and equations of state suggested in the literature. For doing so, 285 experimental data, three equations of state, and two empirical correlations have been checked.

#### Justification by equations of state

This section compares the accuracy of PC-SAFT, SRK, and PR equations of state and the Arrhenius-shape correlation. Literature reports the uncertainty level of these equations of state for monitoring the 2-propanol/hydrogen^39^, furfuryl alcohol/hydrogen^22^, and allyl alcohol/hydrogen^40^ phase equilibria. Table 4 compares the performance of the Arrhenius-shape correlation and equations of state available in the literature^22,39,40^. The bold cells indicate the highest prediction accuracy for each alcohol/hydrogen mixture.

**Table 4:** Validation of the performance of the Arrhenius-shape correlation by PC-SAFT, PR, and SRK.

H_2_ +	Prediction uncertainty in terms of AARD%			
	PC-SAFT	PR	SRK	Arrhenius equation
2-propanol	5.30^39^	7.20^39^	11.30^39^	**2.57**
Furfuryl alcohol	4.62^22^	14.15^22^	14.62^22^	**2.25**
Allyl alcohol	–	3.45^40^	–	**2.46**
Whole data	4.77	9.88	13.89	**2.36**

It can be seen that the Arrhenius-shape correlation has the most reliable results for all alcohol/hydrogen binary solutions. This three-parameter correlation has a simpler shape than the equations of state, needs lower computational effort, and enhances the previously achieved accuracy for hydrogen dissolution in allyl alcohol, furfuryl alcohol, and 2-propanol by at least 28.7%, 51.3%, and 51.5%, respectively. The overall uncertainty in the Arrhenius-shape correlation is also 50.5% lower than the best-obtained result by these equations of state.

#### Validation by previously proposed empirical correlation

There are a couple of empirical correlations for predicting the hydrogen dissolution in methanol, ethanol, 1-propanol, and 2-propanol in the literature^41^. The mathematical formulations of these empirical correlations are shown in Eqs. (13) and (14).

13 $$x_{{_{2} }}^{pred} \, = \,\varepsilon_{1} \, + \,\delta_{1} PT\,$$

14 $$x_{{_{2} }}^{pred} \,\, = \,\varepsilon_{2} \, + \,\delta_{2} \,\left( {T/T_{c} } \right)\left( {P/P_{c} } \right)$$

where $\varepsilon_{1}$, $\delta_{1}$, $\varepsilon_{2}$, and $\delta_{2}$ are the constants of these correlations. The values of these constants for methanol/hydrogen, ethanol/hydrogen, 1-propanol/hydrogen, and 2-propanol/hydrogen are separately presented in Table 5.

**Table 5:** Coefficients of available empirical correlations in the literature for calculating H_2_ solubility in the normal primary alcohols with up to four carbon atoms^41^.

Alcohol	Equation (13)		Equation (14)	
	$\varepsilon_{1}$ (–)	$\delta_{1}$ (1/K MPa)	$\varepsilon_{2}$ (–)	$\delta_{2}$ (–)
Methanol	− 0.002360	0.000010	− 0.002360	0.043372
Ethanol	0.000590	0.000016	0.000594	0.051319
1-propanol	− 0.001151	0.000020	− 0.011510	0.056882
1-butanol	− 0.007290	0.000024	0.007286	0.060148

The uncertainty in the Arrhenius-shape model predictions and the available empirical correlations in the literature for hydrogen dissolution in 1-butanol, 1-propanol, ethanol, and methanol alcohols are reported in Table 6 (the cells having the bold font show the lowest uncertainty).

**Table 6:** Comparing the accuracy of the Arrhenius-shape model by available correlations in the literature for estimating the actual data^36^.

H_2_+	The observed AARD%		
	Equation (13)	Equation (14)	Arrhenius equation
1-Butanol	8.42	8.43^41^	**6.67**
1-Propanol	29.45	5.04^41^	**4.04**
Ethanol	9.58	9.68^41^	**4.19**
Methanol	6.79	**4.12**^41^	6.54
Whole data	15.37	6.42	**5.40**

This table approves that the Arrhenius-shape model has the lowest uncertainty for three binary systems (i.e., 1-butanol/hydrogen, 1-propanol/hydrogen, and ethanol/hydrogen), while Eq. (14) presents the most accurate predictions for methanol/hydrogen system only. Indeed, the Arrhenius-shape model improves the previously achieved accuracy for hydrogen dissolution in ethanol, 1-butanol, and 1-propanol by more than 56.3%, 20.9%, and 19.8%, respectively. Furthermore, it is possible to decrease the cumulative prediction uncertainty of hydrogen dissolution in the methanol, ethanol, 1-butanol, and 1-propanol alcohols by at least 15.9%.

Before here, the reliability of the proposed Arrhenius-shape model has been validated by empirical correlations and equations of state available in the literature. The following section focuses on 285 experimental data for further investigating the reliability of the proposed three-parameter correlation.

#### Validation by laboratory-measurement data

**Figure 1:**
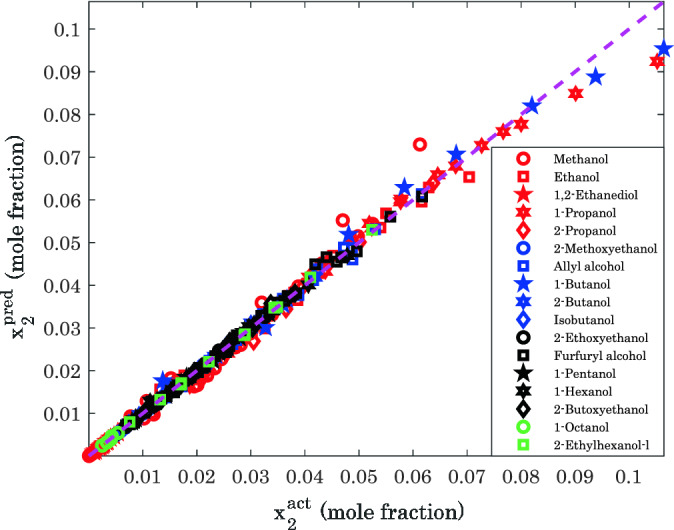
Laboratory versus predicted values of hydrogen solubility in the considered alcohols.

The predicted values of hydrogen solubility in various alcohols by the Arrhenius-shape correlation versus their corresponding actual data are exhibited in Fig. 1. This figure displays predictions versus actual values for all binary alcohol/hydrogen systems. It is not hard to visually approve an excellent agreement between model predictions and experimental data. It is worth noting that the R^2^ of 0.99589 has been observed between the model predictions and laboratory measurements for all alcohol/hydrogen mixtures.

**Figure 2:**
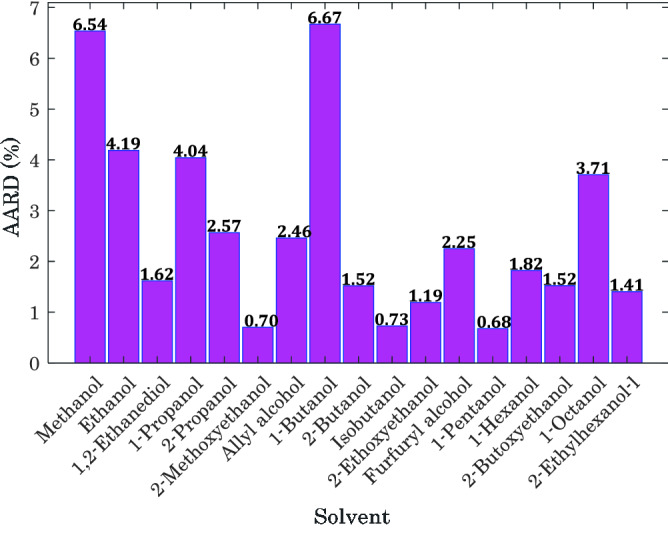
Uncertainty in the prediction of the Arrhenius-shape correlation for various H_2_/alcohol systems.

Figure 2 displays the prediction uncertainty of the Arrhenius-shape correlation for hydrogen solubility in all of the alcohols separately. This figure states that the uncertainty level ranges from AARD = 0.68% (for 1-pentanol/hydrogen) to AARD = 6.67% (for 1-butanol/hydrogen). Furthermore, excluding the phase equilibria of H_2_/methanol and H_2_/1-butanol, all other binary mixtures are simulated with the AARD of lower than 4.19%.

Table 7 utilizes four other statistical matrices (AAE, RAD%, ASE, and R^2^) to investigate the Arrhenius-shape model’s capability for estimating the phase equilibria of seventeen H_2_/alcohol mixtures. The last row of Table 7 presents numerical values of these statistical indices for all alcohol/hydrogen binary systems, i.e., 285 experimental data.

**Table 7:** Assessment the reliability of the Arrhenius-shape correlation using the AAE, RAD%, ASE, and R^2^ indices.

H_2_ (2)+	AAE	RAD%	ASE	R^2^
Methanol	0.0012	8.37	5.44 × 10^–6^	0.99281
Ethanol	0.0014	10.61	3.74 × 10^–6^	0.99320
1,2-Ethanediol	0.0000	4.28	3.52 × 10^–9^	0.99941
1-Propanol	0.0018	8.59	9.58 × 10^–6^	0.99340
2-Propanol	0.0008	7.12	1.80 × 10^–6^	0.99681
2-Methoxy ethanol	0.0001	1.95	9.85 × 10^–9^	0.99995
Allyl alcohol	0.0007	6.58	1.05 × 10^–6^	0.99675
1-Butanol	0.0031	12.51	1.91 × 10^–5^	0.99122
2-Butanol	0.0003	3.18	1.95 × 10^–7^	0.99919
Isobutanol	0.0001	1.28	3.42 × 10^–8^	0.99984
2-Ethoxyethanol	0.0002	2.69	7.50 × 10^–8^	0.99963
Furfuryl alcohol	0.0006	5.08	7.30 × 10^–7^	0.99809
1-Pentanol	0.0002	2.21	6.43 × 10^–8^	0.99952
1-Hexanol	0.0004	3.95	3.06 × 10^–7^	0.99894
2-Butoxyethanol	0.0004	5.41	4.36 × 10^–7^	0.99768
1-Octanol	0.0001	18.11	4.39 × 10^–8^	0.98054
2-Ethyl hexanol-l	0.0003	3.07	1.76 × 10^–7^	0.99958
Whole the data	0.0008	5.51	3.20 × 10^–6^	0.99589

### Monitoring the effect of equilibrium conditions on hydrogen solubility

**Figure 3:**
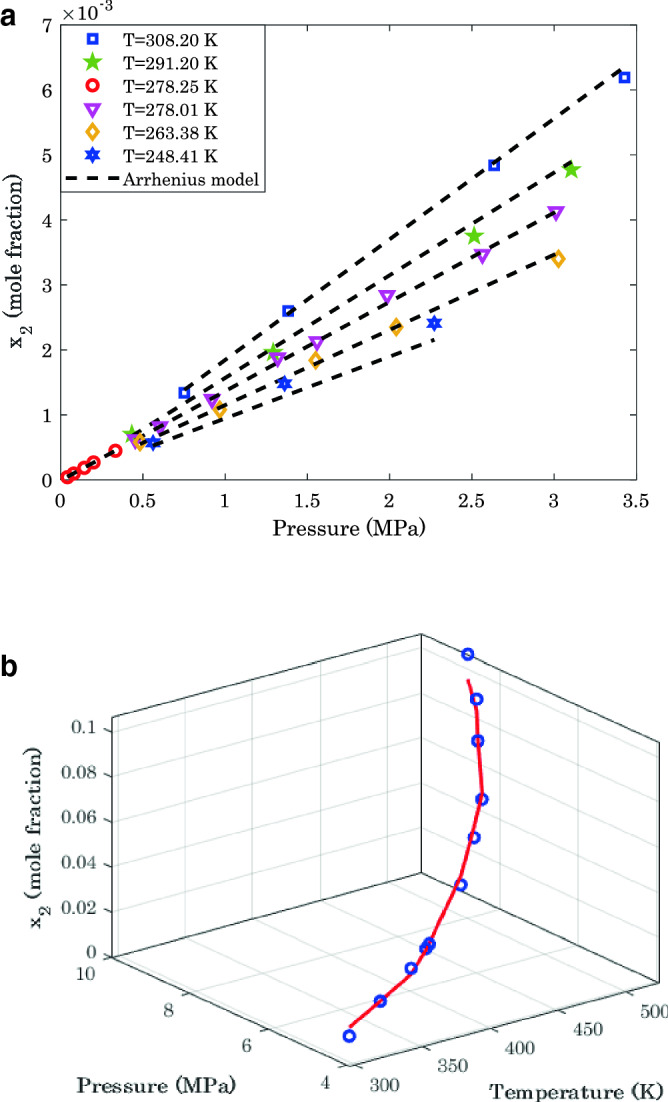
(**a**) Laboratory and modeling results for phase equilibria of methanol/hydrogen. (**b**) Phase equilibria of 1-butanol/hydrogen: laboratory data (**o**) and model prediction (**—**).

It is previously explained that the Arrhenius-shape correlation has the highest uncertainty for H_2_/1-butanol and H_2_/methanol (see Fig. 2). To demonstrate that this level of uncertainty is entirely unimportant, the experimental and modeling results for these two mixtures have been shown in Fig. 3a,b.

Figure 3a represents the effect of pressure on the hydrogen solubility in methanol for six temperature levels. It can be seen that an excellent agreement exists between experimental measurements and predicted values by the Arrhenius-shape correlation. The proposed model correctly persuades the experimental trend and anticipates all individual data points precisely.

Moreover, experimental data and modeling predictions show that the hydrogen absorption capacity of the methanol increases by an increase in either pressure or temperature. This observation was also previously anticipated by the positive values of the Arrhenius correlation ($\alpha$ and $\lambda$). The positive effect of the pressure on the hydrogen solubility may be related to enhancing the mass transfer driving force by increasing the pressure. Therefore, the solubility of gases in liquids (hydrogen solubility in methanol) improves by increasing the pressure. On the other hand, the literature states that increasing the temperature increases the dissolution tendency of a low-soluble gas in the liquids^23^.

Experimental measurements of H_2_ solubility in 1-butanol and their related predictions by the proposed three-parameter model have been presented in Fig. 3b.

Since the experimental measurements for this binary mixture are fully scattered, it is impossible to depict this graph on a two-dimensional scale. Meanwhile, excellent compatibility can be understood between experimental and modeling findings. Furthermore, like the H_2_/methanol mixture, the pressure and temperature positively affect the hydrogen absorption capacity of 1-butanol. All reasons for explaining the impact of temperature and pressure on the H_2_/methanol phase equilibria may also be referred to here.

### Monitoring the effect of alcohol type on the hydrogen solubility

**Figure 4:**
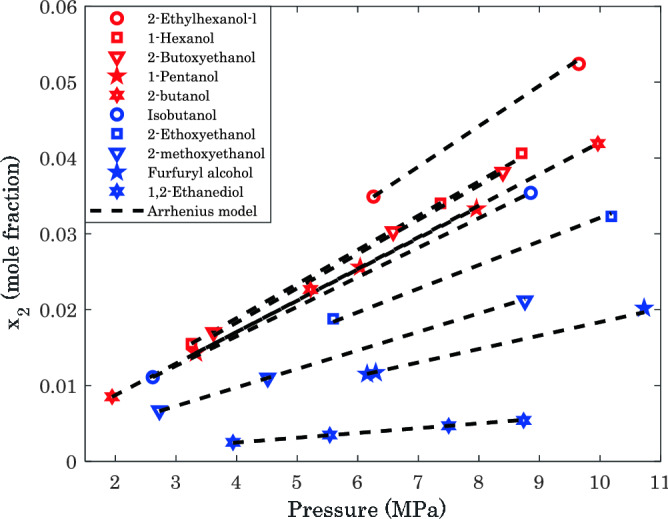
Comparing the H_2_ capture capacity of several alcohols at T = 373.15 K.

Figure 4 is plotted to check the hydrogen capture capacity of the investigated alcohols. Since the reported equilibrium conditions for some of the H_2_/alcohol systems are fully scattered, it is impossible to compare all seventeen binary mixtures. Therefore, Fig. 4 only compares the hydrogen absorption capacity of ten alcohol/hydrogen mixtures using experimental data and modeling predictions. Visual inspection can easily approve the excellent compatibility between experimental and modeling data. Moreover, the AARDs between actual data and modeling results for all cases are lower than 1.27%.

The positive impact of temperature and pressure on the H_2_ absorption tendency of these alcohols is also quite clear. Moreover, this analysis approves that 2-ethyl hexanol has the highest hydrogen absorption capacity among the ten investigated alcoholic media at T = 373.15 K.

### Pure simulation results

Since the actual measurements of phase equilibria of some alcohol/hydrogen systems are fully scattered, and their temperature and pressure are different, it is impossible to make a comparison only using the experimental data. Therefore, this section relies on the Arrhenius-shape correlation to make the comparison on an identical basis. The comparisons are made on the average hydrogen capture capacity of the investigated alcohols and the impact of operating conditions on hydrogen dissolution. This comparison analysis helps find the alcohol with the maximum ability to capture H_2_ molecules.

#### Tracking the effect of alcohol types

**Figure 5:**
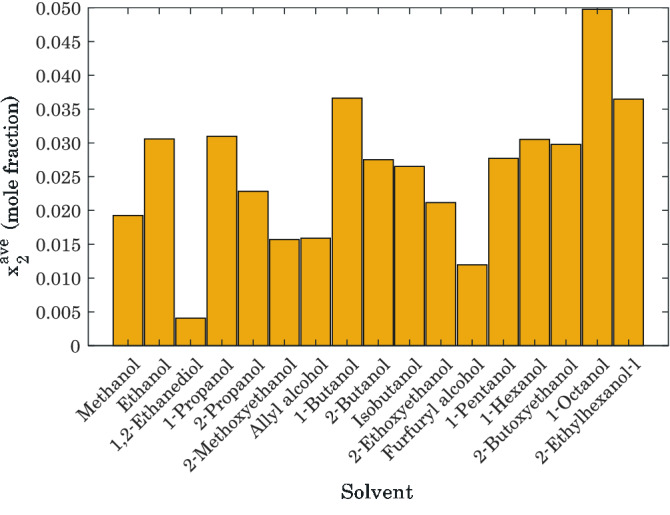
Average values of hydrogen absorption capacity of seventeen alcohols in the range of 5.197 < P < 8.82 and 341.5 < T < 373.15: pure simulation results.

Investigating the experimental databank (see Table 1) indicates that 5.197 < P < 8.82 MPa and 341.5 < T < 373.15 K is utilized for measuring the hydrogen dissolution in all seventeen checked alcohols. For comparison on the same basis, this joint operating range is fed into the Arrhenius-shape correlation to calculate the average values of hydrogen dissolution in various alcohols (Fig. 5). It can be readily observed that 1-octanol and 1,2-ethanediol have the highest and lowest tendency to absorb hydrogen molecules, respectively.

#### Monitoring the effect of operating conditions

**Figure 6:**
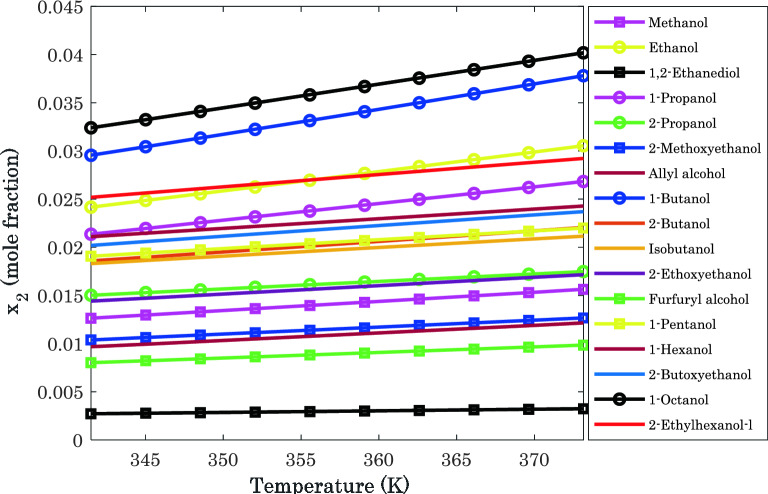
Comparing the H_2_ capturing ability of the considered alcohols at P = 5.197 MPa: pure simulation results.

Simulation profiles for hydrogen dissolution in different alcohols versus temperature at P = 5.197 MPa have been shown in Fig. 6. This figure also approves that 1-octanol and 1,2-ethanediol have the highest and lowest tendency for hydrogen absorption in a wide range of temperatures.

**Figure 7:**
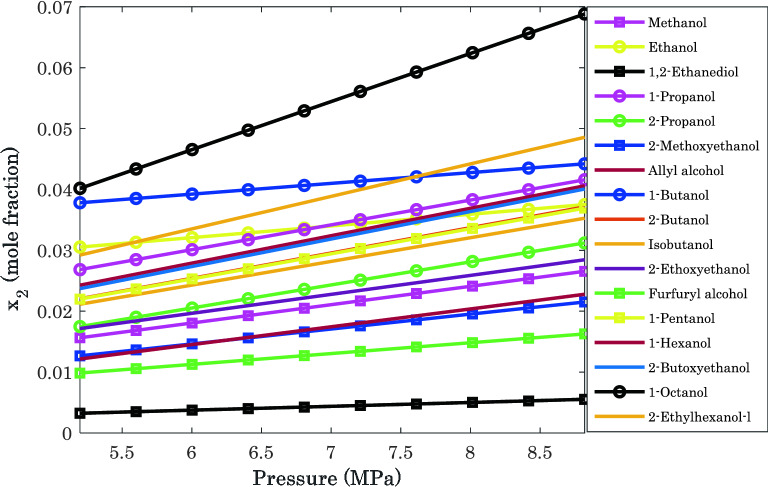
Investigating the H_2_ capturing ability of the considered alcohols at T = 373.15 K: pure simulation results.

The prediction results for hydrogen dissolution in different alcohols versus pressure at T = 373.15 K have been presented in Fig. 7. Like the previous findings, it is clear that 1-octanol and 1,2-ethanediol have the highest and lowest ability to absorb H_2_ molecules from a gas stream.

A deep investigation of the experimental and simulation results in Figs. 4 and 7 justifies that the phase equilibria of all H_2_/alcohol solutions obey Henry’s law (Eq. 15) for almost all pressure and temperature conditions.

15 $$P_{{_{2} }} \, = \,x_{{_{2} }} \, \times \,H_{L}$$

In Eq. (15), $P_{{_{2} }}$ is the H_2_ partial pressure in gas phase, $x_{{_{2} }}$ shows H_2_ mole fraction in alcohols, and H_L_ indicates Henry’s law constant.

### Graphical analysis for maximizing hydrogen solubility in 1-octanol

**Figure 8:**
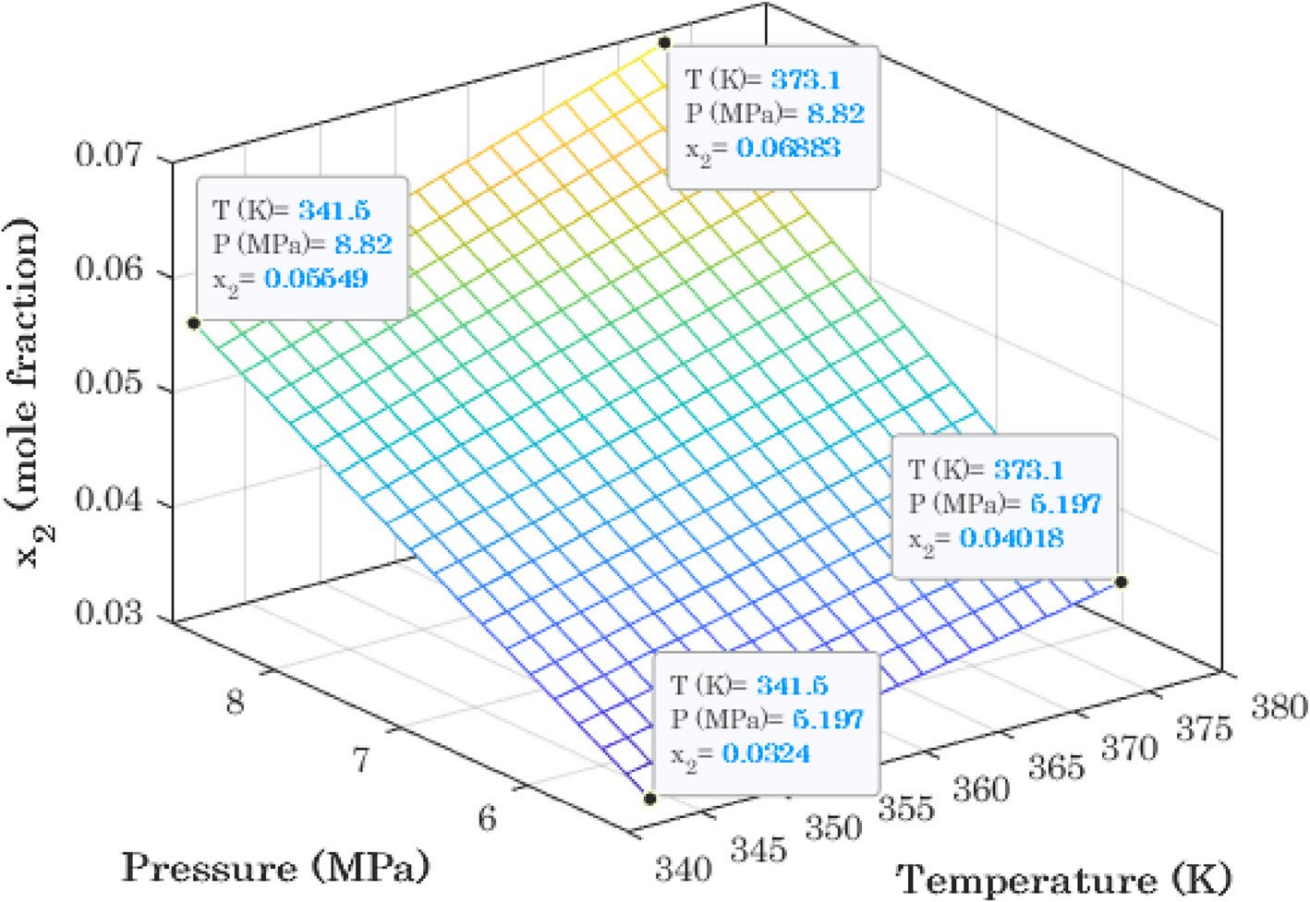
Three-dimensional profile for phase equilibria of 1-octanol/hydrogen binary mixture.

Three previous simulation graphs predicted that 1-octanol has the maximum ability to capture H_2_ molecules. Therefore, the surface (3D) profile of hydrogen dissolution in 1-octanol versus temperature and pressure is simulated using the Arrhenius-shape correlation (Fig. 8). This type of simulation graph helps determine equilibrium conditions that maximize H_2_ dissolution in alcohol. Figure 8 states that the highest hydrogen dissolution of 0.06883 can be achieved at the maximum allowable operating conditions (T = 373.1 K and P = 8.82 MPa). This figure also shows that it is possible to double hydrogen solubility in 1-octanol by increasing temperature from 341.5 K to 373.1 K (~ 32 K) and pressure from 5.197 MPa to 8.82 MPa (~ 3.6 MPa).

## Conclusion

This study established a general three-parameter correlation for accurately predicting the hydrogen dissolution in seventeen alcohols (i.e., methanol, ethanol, 1,2-ethanediol, 1-propanol, 2-propanol, 2-methoxy ethanol, allyl alcohol, 1-butanol, 2-butanol, isobutanol, 2-ethoxyethanol, furfuryl alcohol, 1-pentanol, 1-hexanol, 2-butoxyethanol, 1-octanol, and 2-ethyl hexanol-l.). Indeed, the pre-exponential and exponential parts of this Arrhenius-shape correlation were related to the pressure and temperature, respectively. This model not only has the simplest possible form, but it is also the most generalized/accurate correlation for phase equilibrium monitoring of alcohol/hydrogen binary systems. Only the phase equilibria of methanol/hydrogen and 1-butanol/hydrogen have been simulated with the AARD of higher than 6%, and all other binary mixtures have the AARD of lower than 4.2%. The proposed correlation estimates whole of the experimental solubility data with the AARD = 3.28%, AAE = 8 × 10^–4^, RAD = 5.51%, ASE = 3.20 × 10^–6^, and R^2^ = 0.99589. Moreover, the Arrhenius-shape model has more accurate predictions than the equations of state and empirical correlations in the literature. The pure simulation analysis exhibited that 1-octanol and 1,2-ethanediol have the highest and lowest tendency to absorb the hydrogen molecules. Furthermore, it was also observed that all alcohol/hydrogen binary mixtures thermodynamically obey Henry’s law. The maximum hydrogen dissolution of 0.06883 can be reached at the maximum allowable operating pressure and temperature (T = 373.1 K and P = 8.82 MPa).

## Data Availability

All data generated or analyzed during this study are available on reasonable request from the corresponding author.
